# Boundary Associated Long Noncoding RNA Mediates Long-Range Chromosomal Interactions

**DOI:** 10.1371/journal.pone.0136104

**Published:** 2015-08-24

**Authors:** Ifeoma Jane Nwigwe, Yoon Jung Kim, David A. Wacker, Tae Hoon Kim

**Affiliations:** 1 Department of Molecular Biophysics and Biochemistry, Yale University School of Medicine, New Haven, CT, 06520, United States of America; 2 Center for Systems Biology and Department of Biological Sciences, The University of Texas at Dallas, Richardson, TX, 75080, United States of America; 3 Departments of Internal and Emergency Medicine, University of Maryland School of Medicine, Baltimore, MD, 21201, United States of America; Florida State University, UNITED STATES

## Abstract

CCCTC binding factor (CTCF) is involved in organizing chromosomes into mega base-sized, topologically associated domains (TADs) along with other factors that define sub-TAD organization. CTCF-Cohesin interactions have been shown to be critical for transcription insulation activity as it stabilizes long-range interactions to promote proper gene expression. Previous studies suggest that heterochromatin boundary activity of CTCF may be independent of Cohesin, and there may be additional mechanisms for defining topological domains. Here, we show that a boundary site we previously identified known as CTCF binding site 5 (CBS5) from the homeotic gene cluster A (HOXA) locus exhibits robust promoter activity. This promoter activity from the CBS5 boundary element generates a long noncoding RNA that we designate as *b*oundary associated *l*ong *n*oncoding *RNA-1* (blncRNA1). Functional characterization of this RNA suggests that the transcript stabilizes long-range interactions at the HOXA locus and promotes proper expression of HOXA genes. Additionally, our functional analysis also shows that this RNA is not needed in the stabilization of CTCF-Cohesin interactions however CTCF-Cohesin interactions are critical in the transcription of blncRNA1. Thus, the CTCF-associated boundary element, CBS5, employs both Cohesin and noncoding RNA to establish and maintain topologically associated domains at the HOXA locus.

## Introduction

CCCTC binding factor (CTCF) is an eleven zinc finger protein that affects gene expression by a variety of mechanisms [1–3]. Initially, CTCF was characterized as a transcription repressor of the c-Myc promoter [4]. Later studies have shown that CTCF can also act as a transcription activator [5]. CTCF regulates transcription of the XIST gene and RNA interactions made with CTCF are critical for X chromosome inactivation (XCI) [6–8]. Notably, Cohesin has been found to bind to a majority of CTCF binding sites (CBSs) across the genome [9]. CTCF-Cohesin interactions are critical to the function of CTCF [2, 9, 10]. CTCF recruits Cohesin directly via Cohesin subunit SA1/SA2 to facilitate long-range interactions [9]. The long-range interactions made by CTCF are critical for transcription insulation. Most recently, CTCF has been recognized as a critical component responsible for chromosome looping and topological organization of the genome [11–13].

Transcription insulation activities of CTCF include enhancer-blocking and heterochromatin boundary activities are two distinct assayable properties linked to a subclass of CBSs. Enhancer-blocking function resulting from CTCF–Cohesin interactions have been best characterized at the H19/Igf2 locus and the β-globin locus [2]. In contrast, CTCF heterochromatin boundary activity is less well characterized, but may be independent of Cohesin [14–16]. Heterochromatin boundary activity was first characterized in *Sacchromyces cerevisiae*’s HMR locus and has been linked to Transcription Factor III (TFIII) C (TFIIIC) and RNA Polymerase (RNAP) III (RNAPIII) activity at the promoter region of the tRNA^Thr^ gene encoded at the HMR locus [17, 18]. Studies in yeast have shown that recruitment of RNAP can disrupt or antagonize heterochromatin [17–20]. In higher eukaryotes boundary activity has yet to be linked to active RNAP transcription, although it has been suggested to be associated with topological boundaries in the human genome [21]. In contrast, stalled promoters in *Drosophila*, have been shown to confer boundary activity along the HOX locus [22]. Interestingly, the insulator protein CTCF has been shown to interact with RNAPII in eukaryotes [23]. However, it remains unclear if active promoters might function at heterochromatin boundaries in the human genome and whether RNAPII transcription at boundaries generates functional noncoding RNAs (ncRNAs) [21].

There is growing evidence that noncoding RNAs play a significant role in long-range interactions mediating interactions between and within [8, 24–29]. The role of RNAs in the structural organization of the genome has been suggested years ago in a study showing that RNAs are critical to the integrity of the nuclear matrix [29]. Since then, transcripts like XIST [8, 12, 28, 30], Steroid receptor RNA activator (SRA) long noncoding RNA (lncRNA) [25], Kcqn1ot1 [26], enhancer RNAs (eRNAs) [31], and Functional intergenic repeating RNA element lncRNA [27] have been shown that long noncoding RNAs have a structure role in the integrity of TDs and the expression of genes at specific genomic loci. Unlike Cohesin interactions made with CTCF, it is not clear how lncRNAs coordinate long-range chromosomal interactions and regulate gene expression. Studies with SRA lncRNA and Kcqn1ot1 suggest that these transcripts either stabilize CTCF-Cohesin interactions or interact directly at its genomic loci target to mediate long-range interactions needed to maintain the genome architecture and regulate gene expression. SRA lncRNA acts in *trans* at the H19/Igf2 locus to facilitate a long-range interaction mediated by CTCF and Cohesin. RNA-DEAD box helicase p68 interacts with SRA and facilitate CTCF-Cohesin interactions, which in turn stabilize the chromosomal loop at the H19/Igf2 locus [25]. In more recent studies, lncRNAs have been shown to interact directly with CTCF [8, 32]. Interactions made directly with CTCF may occur at the C-terminal domain of CTCF [8] or the recently identified RNA binding domain of CTCF [32]. Based on what is known about SRA interactions with CTCF and its effect on long-range interactions, it has been hypothesized that CTCF-RNA interactions may be critical for topological organization of the genome. In addition, XCI studies have shown that CTCF-RNA interactions can act in *cis* or *trans* to regulate gene expression and genome topology [6, 8, 12, 30]. Alternative to CTCF-RNA interactions, Kcqn1ot1 has shown that long-range interactions can be mediated through direct RNA-DNA interactions, independent of CTCF and Cohesin. Lastly, eRNAs have been shown to be important for long-range interactions that mediate promoter-enhancer communications [31]. Thus noncoding RNAs represent a growing class of macromolecules capable of organizing chromosome and mediating gene regulatory programs.

We sought to explore the relationships among CTCF-RNAPII interactions and noncoding RNA at a heterochromatin boundary element in the HOXA locus. Specifically, we were interested in the potential role(s) that CTCF-RNAPII interactions may play in topological organization at the HOXA boundary element, referred to as CBS5. We have detected reproducible RNAPII occupancy at CBS5, and show that these interactions are imperative for robust promoter activity in reporter assays. CBS5 promoter activity is also critical for the generation of a noncoding transcript in vivo across many cell types. We clone and characterize the corresponding full-length noncoding RNA that we referred to as boundary associated long noncoding RNA-1 (blncRNA1). We investigated the function of the blncRNA1 by knockdowns. Our experiments show that this transcript mediates long-range interactions at the HOXA locus that seems independent of CTCF-Cohesin interactions. Thus, our results suggest that both Cohesin and noncoding RNA at a boundary element can facilitate long-range chromosomal interactions.

## Materials & Methods

### Cell Lines and Antibodies

Fetal lung fibroblast (IMR90), and foreskin fibroblast were obtained from American Type Culture Collection and grown as directed by the supplier. CTCF and Rad21 antibodies were obtained from Millipore; H3K27me3 and RNAPII antibodies were purchased from Abcam.

### Constructs

Promoter activity constructs were cloned into pGL3-Enhancer Vector (Promega, Madison, WI). To normalize luciferase activity pRL-CMV was used as the control renilla plasmid according to Dual-Luciferase Reporter Assay System (Promega, Madison, WI). Primer sequences used to clone the promoter can be found in S1 File.

### Luciferase Assay

All transfections were carried out using the rapid 96-well format. Cell extracts were prepared and analyzed 48hrs later according to Dual-Luciferase Reporter Assay System (Promega, Madison, WI). To monitor the promoter activity a total of 6x10^4^ cells were transfected with 0.5 μL of lipofectamine-2000, 300ng of reporter construct, and 1 ng of the control renilla plasmid.

### Rapid Amplification of cDNA Ends (RACE)

Isolation of blncRNA1 at CBS5 was isolated using 5’ and 3’ RACE according to First Choice RLM RACE Kit (Ambion, Carlsbad, CA) guidelines. 5’ and 3’ inner and outer primer sequences for nested PCR are located in S1 File.

### blncRNA1 Expression Analysis

Total RNA was extracted using Trizol (Life Technologies, Carlsbad, CA). The extracted RNA was treated with Roche’s DNAseI, phenol-chloroform extracted, and precipitated with sodium acetate and ethanol. Polyadenlyated transcripts were selected using oligo-dT during cDNA synthesis with Invitrogen’s Superscript III (Life Technologies, Carlsbad, CA). The cDNA was then treated with RNAse and purified using Qiaquick PCR Purification Kit (Qiagen, Germany). blncRNA1 expression was detected by reverse transcription coupled to quantitative real-time PCR (RT-qPCR). Detection primer sequences are in S1 File.

### Lentivirus Transduction

blncRNA1 shRNA lentivirus knockdown plasmids were cloned into Invitrogen’s pLenti6.4/R4R2/V5-DEST as instructed by the manufacturer. Invitrogen lentivirus plasmids were cotransfected with Viral Power in HEK293T cells. IMR90s were infected with shRNA lentiviruses at an MOI3, and MOI5 was used for the overexpression of the lentivirus. Primer sequences for construction of blncRNA1 shRNA lentiviruses are provided in S1 File. CTCF and Rad21 knockdown viruses were prepared as previously described [14].

### Chromatin Immunoprecipitation (ChIP)

ChIP was performed using 1 mg of chromatin as previously described. ChIP-qPCR was performed using 500pg of the purified ChIP DNA as previously described [14]. ChIP-qPCR primer sequences are in S1 File.

### Chromosome Capture Confirmation (3C)

3C was performed as previously described [14, 33–35]. To monitor interactions at the HOXA locus upon knockdown of blncRNA we used an shRNA control in the lentivirus transduction that has no known targets in the genome. Upstream and downstream coordinates tested in the 3C assay were previously described [14]. Interaction frequencies were calculated as previously described [14, 33–35]. 3C primer sequences are in S1 File.

## Results

### CBS5 Boundary Element Is a Robust Promoter

**Fig 1:**
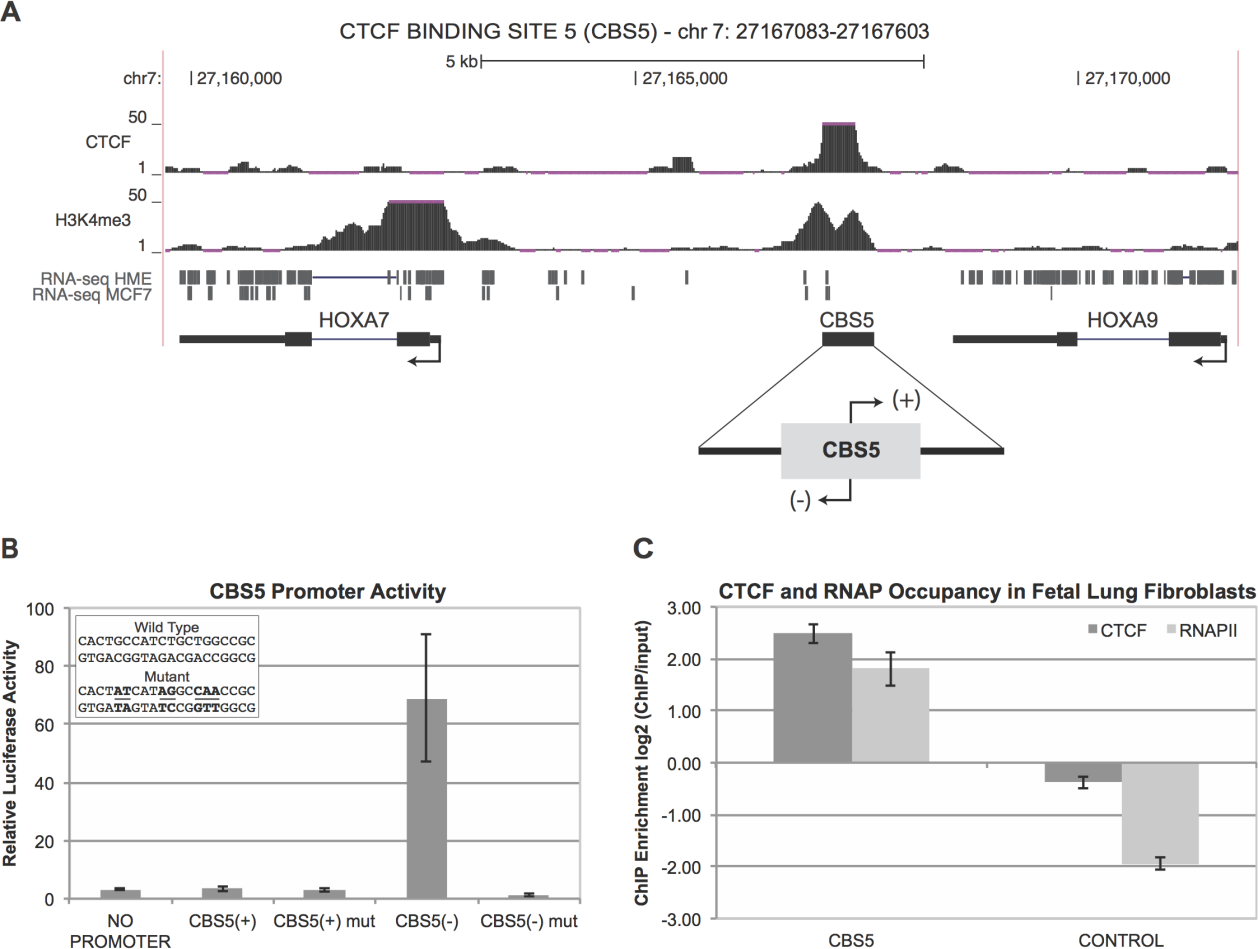
HOXA barrier CBS5 is a promoter. (A) Lung fibroblast CTCF and H3K4me3 ChIP signals from the UCSC genome browser are shown (hg18 build). CBS5 is located between HOXA7 and HOXA9 (chr7:27167083–27167603). HME and MCF7 RNAseq tags from the UCSC genome browser align to CBS5. These tags were used for isolation of the blncRNA1 by 5’ and 3’ RACE. CBS5 was cloned upstream of the luciferase reporter gene in pGL3 SV40 Enhancer Vector. CBS5 fragment was inserted either antisense CBS5 (+) or sense CBS5 (-) relative to the hg18 assembly of the human genome. (B) CBS5 Promoter Activity. Activity of the CBS5 reporter constructs were measured and normalized to the luciferase activity detected for a positive control, the SV40 Promoter/Enhancer reporter. The y-axis represents the percentage of CBS5 reporter activity normalized to the SV40 Promoter/Enhancer. The inset denotes the wild type CTCF core binding sequence and the mutant core binding sequence in CBS5(-)mut. The specific mutations made are underlined in the wild type and mutant core sequence. (C) CTCF-RNAPII interactions in fetal lung fibroblast. ChIP-qPCR results from CTCF and RNAPII in fetal lung fibroblast. The y-axis represents enrichment of bound protein normalized to ChIP input and is in a base 2 logarithmic scale. The control represents a motif matched CBS where CTCF has been shown not to bind [14].

Our initial characterization of boundary activity at the HOXA locus on chromosome 7 started with reporter assays to determine if the HOXA boundary element, CBS5, exhibited any other activities besides the boundary activity that we reported previously. We cloned the 591bp fragment containing CBS5 into the promoter region of pGL3 luciferase vector that also contained SV40 enhancer element (Fig 1A). CBS5 sequence was cloned either CBS5 (-) or CBS5 (+) orientations relative to the genome hg18 build from the UCSC genome browser [36]. Due to low transfection efficiency of primary fibroblasts, we performed our reporter assays in HEK293 cells. When CBS5 is cloned in the same orientation as the other HOXA genes (HOXA7 and HOXA9), CBS5 (-), the reporter exhibited 70% of the promoter activity observed from the SV40 Promoter/Enhancer control reporter (Fig 1B). In contrast, CBS5 cloned in the opposite orientation, CBS5 (+), displayed only 5% activity. This result suggests that CBS5 drives transcription on the (-) strand. The direction of CBS5 promoter activity is orientated similar to the transcription of HOXA genes in the locus. To test the specificity of the detected promoter activity we made mutations within CTCF’s consensus sequence that has been previously identified [37] and monitored its effect on the reporter activity. Mutations, CBS5 (-) mut made within the CTCF’s motif (Fig 1B) in the CBS5 (-) construct, drastically reduce the promoter activity. A marginal change in promoter activity was detected when we mutated CTCF’s core in the CBS5 (+) mut construct (Fig 1B).

**Fig 2:**
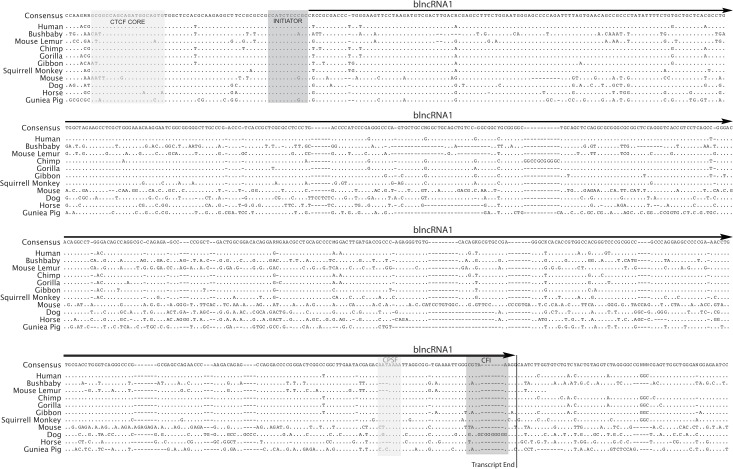
Cloning and annotation of blncRNA1. Sequence alignment of the cloned full-length blncRNA1 shows that CTCF core binding sequence (CTCF core) and the predicted initiator element are conserved. The sequence alignment was performed using Geneious Pro 5.46 ClustalW alignment feature (http://www.genious.com, [40]). The consensus sequence of blncRNA1 is shown where dots represents conservation of nucleotide, dashes represent gaps in the species tested, and the isolated transcript is outlined by the arrow. The conserved features in blncRNA1 are outlined in the sequence alignment. The 3’ alignment shows conservation of sequence elements critical for transcription termination; these factors cleavage/polyadenylation specificity factor (CPSF) and cleavage factor I (CFI) sites are noted within the alignment.

ClustalW sequence alignment of of HOXA CBS5 revealed an initiator element [38] conserved in mammals with a 95.7% pairwise identity (Fig 2). To confirm the identity of the initiator required for RNAPII transcription at CBS5, we mutated the conserved motif in the CBS5 (-) reporter and monitored the effect of these mutations by reporter assay (S1 Fig). Mutations made to the initiator element resulted in a 42% reduction of the luciferase activity from CBS5 (S1 and S2 Figs). These results suggest that CBS5 exhibits robust promoter activity that is dependent on CTCF motif and initiator element.

The reporter assays suggested that CBS5 exhibits promoter activity and may recruit RNAPII if this activity is relevant at the endogenous site. We analyzed the endogenous CBS5 for CTCF-RNAPII interactions by chromatin immunoprecipitation (ChIP) coupled to qPCR (ChIP-qPCR) assay in fetal lung fibroblasts. The ChIP-qPCR data showed enrichment for RNAPII at CBS5 (Fig 1C, S1 File), which supports our hypothesis of RNAPII recruitment to CBS5 by CTCF.

A previous study showed that a single CBS promotes reporter expression and the activity is the result of CTCF-RNAPII interactions [23]. Furthermore, it was shown that not all CBS exhibit CTCF-RNAPII recruitment and as a result may not promote reporter expression [23]. Our findings provide further support to the observation that a single CBS can drive reporter expression.

### Isolation and Detection of blncRNA1 from CBS5

RNAPII interactions at CBS5 and the associated promoter activity suggested to us that a noncoding transcript might be generated from CBS5. The direction of the promoter activity guided our attempts to isolate this noncoding transcript by 5’ and 3’ RACE. Further support of the transcript came from Christopher Burge’s RNAseq data [39]. This data set contained several RNA-seq tags from human mammary epithelial cells (HMECs) and MCF7 breast cancer cells that aligned to the suspected start of the noncoding transcript (Fig 1A). To isolate the potential transcript from CBS5 we used the sequence tags as anchors for our 3’ and 5’ rapid amplification of cDNA ends (RACE) analyses. Our efforts resulted in successful isolation of a 572nt single exon polyadenylated transcript from CBS5 in HEK293. Comparative sequence analysis using ClustalW sequence alignment on Geneious Pro 5.4.6 (http://genious.com, [40]) revealed that the noncoding transcript is highly conserved at the 5’ end in mammals (Fig 2). The 3’ end of the transcript that contains the cleavage/polyadenylation specific factor (CPSF) [41] site and cleavage factor I (CFI) site [41] needed for polyadenylation and both sites exhibit sequence conservation (Fig 2). Using the full-length sequence of blncRNA1, we designed primers for reverse transcription (RT) coupled quantitative PCR (qPCR) detection. Using these primers, we have detected the expression of blncRNA1 in human diploid fetal lung fibroblasts, thus confirming expression of blncRNA1 in HEK293 and IMR90 cells.

### blncRNA1 Is Expressed in Differentiated Cells and Not in Stem Cells

**Fig 3:**
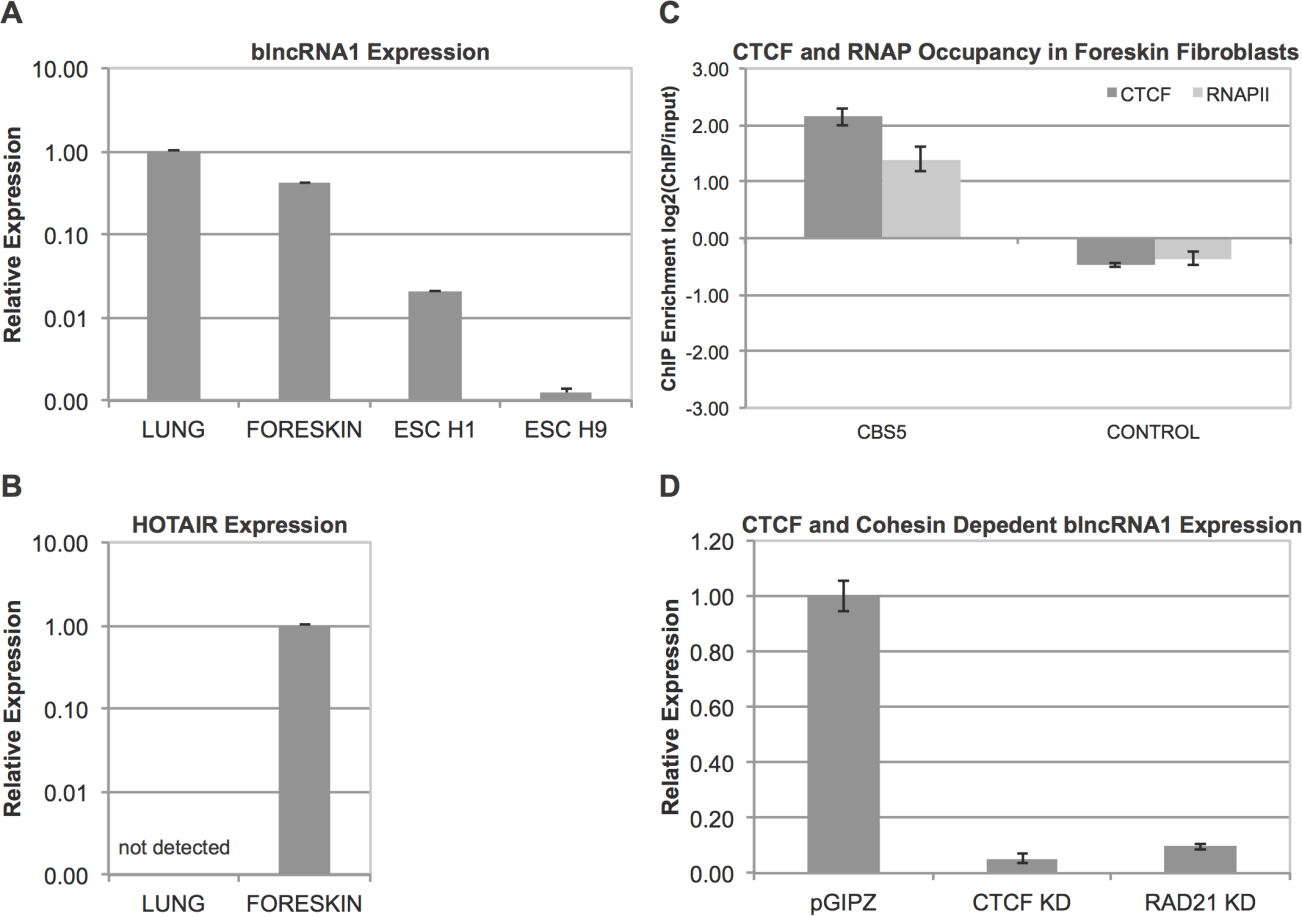
blncRNA1 Expression Analysis. (A) blncRNA1 detection in human ESC. blncRNA1 expression was tested in H1 (male) and H9 (female) hESC. The expression was normalized to expression in fetal lung fibroblast. (B) blncRNA1 detection in anterior and posterior fibroblast. blncRNA1 expression was tested in anterior (fetal lung) and posterior (foreskin) fibroblast. HOTAIR expression control was used to distinguish expression pattern of anterior and posterior fibroblast HOX genes. HOTAIR expression was normalized to foreskin fibroblast. blncRNA1 expression was normalized to the expression in fetal lung fibroblast. (C) CTCF-RNAPII interactions in posterior fibroblast. CTCF and RNAPII ChIP-qPCR were performed in foreskin fibroblast. The y-axis represents enrichment of bound protein normalized to ChIP input. The control represents a motif matched CBS where CTCF has been shown not to bind [14]. (D) blncRNA1 expression is dependent on CTCF and Cohesin. blncRNA1 expression levels were determined by RT-qPCR after infection with empty pGIPZ lentiviral particles or lentiviral particles coding shRNAs against CTCF or RAD21 in fetal lung fibroblast. The y-axis represents blncRNA1 expression normalized to the control (pGIPZ).

Upon confirming expression of blncRNA1 in fetal lung fibroblasts, we checked if the transcript is expressed in other cell lines, in particular, human embryonic stem cells (hESC) and other fibroblasts. We used H1 and H9 ESC to check the expression of the blncRNA1 transcript by RT-qPCR. The transcript was barely detectable in H1 and H9 ESC (less than 0.1% of the level found in fetal lung fibroblasts, Fig 3A, S1 File). Since blncRNA1 was detected in anteriorly derived fetal lung fibroblasts, we were curious if the transcript is also expressed in more posteriorly derived fibroblasts whose heterochromatin (mainly methylation of histone H3 at lysine 27 residue, H3K27me3) deposition pattern is inverse of the fetal lung fibroblast. We analyzed CTCF and RNAPII interactions at CBS5 by ChIP-qPCR in posterior fibroblasts and observed that similar interactions are made at CBS5 in foreskin fibroblasts (Fig 3, S1 File). We also detected these interactions in leg, butt, and butt-thigh fibroblasts derived from the posterior axis (Figures A-C in S2 Fig, S1 File). The expression of blncRNA1 was monitored by RT-qPCR in these fibroblasts. As a control, we used HOTAIR, a noncoding RNA that is expressed posteriorly. We detected blncRNA1 in both foreskin and fetal lung fibroblasts, but HOTAIR was only expressed in foreskin fibroblasts as expected (Fig 3A and 3B, S1 File). We also checked blncRNA1 and HOTAIR expression additional primary fibroblasts derived lines from leg, butt, and butt-thigh (Figures D-E in S2 Fig, S1 File) and found that blncRNA1 was expressed in all posterior fibroblasts analyzed [42]. Detection of this transcript in differentiated fibroblasts and not ESCs suggest that blncRNA1 may function in a capacity that is important to differentiated cells.

### blncRNA1 Expression Is Dependent on CTCF and Cohesin

Luciferase reporter assays showed that CBS5 promoter activity is lost upon mutations of the CTCF motif and initiator element at the transcription start site. We wanted to confirm that the expression of this transcript from the endogenous locus is dependent on CTCF. Using lentiviral particles expressing shRNA against CTCF we performed CTCF or Cohesin subunit RAD21 knockdown and analyzed blncRNA1 expression by RT-qPCR. We detected reduction of blncRNA1 expression upon the knockdown of either CTCF or RAD21 but not with the pGIPZ control (Fig 3D, S1 File). The loss of blncRNA1 expression upon CTCF knockdown is consistent with the reporter assay results (Fig 1C, S1 File). These results show that CTCF at CBS5 is critical for recruitment of RNAPII to drive the expression of blncRNA1. In addition, CTCF-dependent expression of blncRNA1 is consistent with a previous study concerning the Wrap53 noncoding RNA whose expression is reduced upon the loss of CTCF [32].

### blncRNA1 Knockdown Results in Alteration of HOXA7 Expression

**Fig 4:**
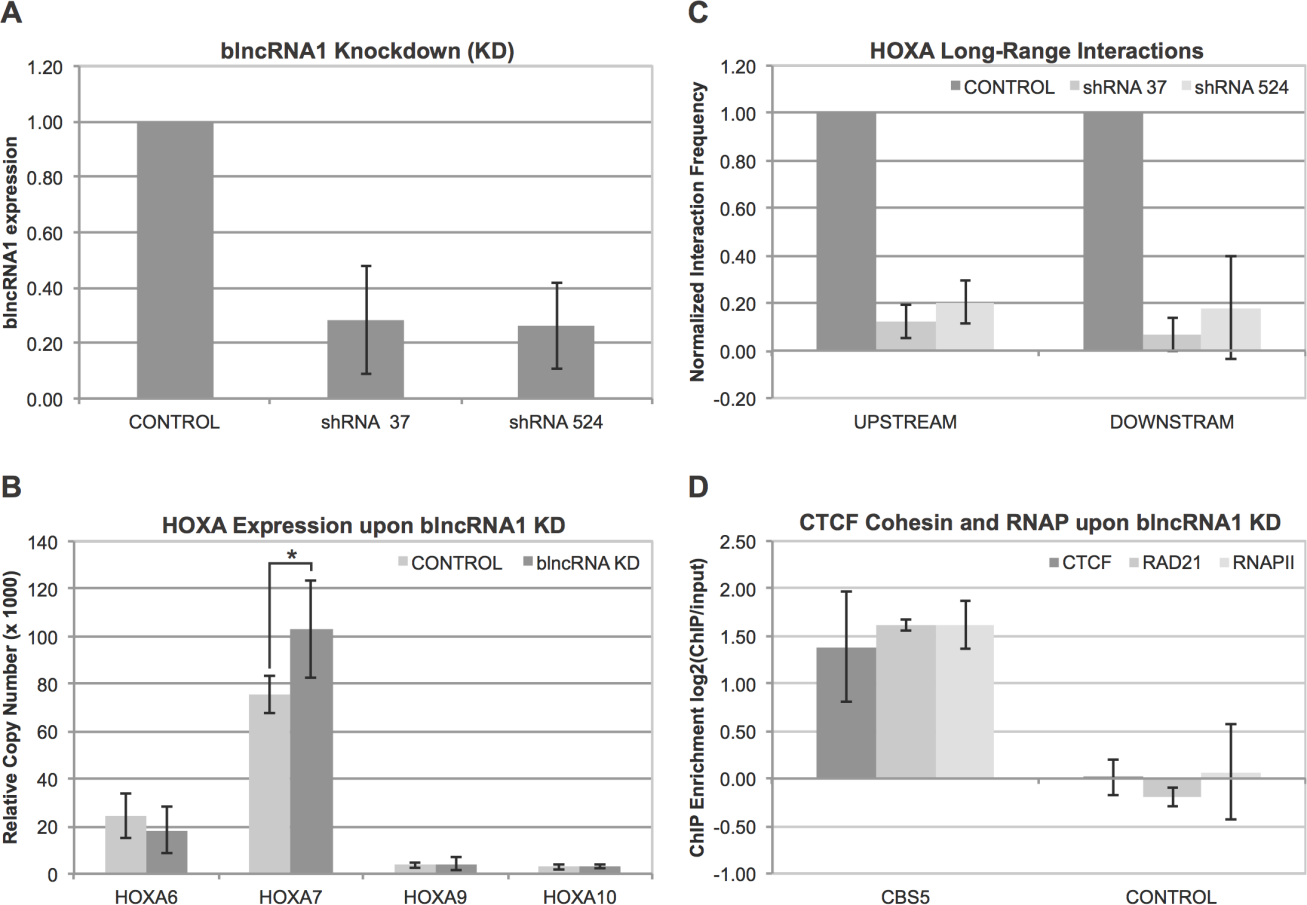
blncRNA1 function at HOXA locus. (A) blncRNA1 knockdown in fetal lung fibroblast. 5’ and 3’ lentiviruses expressing 5’ and 3’ shRNA target were used to knockdown blncRNA1. Control was an empty pGIPZ lentiviral vector. Successful knockdown of blncRNA1 was confirmed by qRT-PCR. The y-axis represents normalized blncRNA1 expression to mock shRNA knockdown. (B) Expression pattern of HOXA genes near CBS5 upon blncRNA1 knockdown. The y-axis represents relative copy number of cDNA for each HOXA gene analyzed by realtime quantitative PCR. Only HOXA7 gene showed a significant (*P* = 0.048, T-test) increase in expression upon blncRNA1 knockdown. All other HOXA genes showed no significant effect. (C) 3C analysis of long-range interactions on the HOXA locus. In this assay we focused on upstream and down stream interactions upon shRNA knockdown at the 5’ or 3’ end of blncRNA1. The y-axis represents interaction frequencies normalized to mock shRNA knockdown. (D) CTCF-Cohesin-RNAP interactions at CBS5 upon 3’ target shRNA knockdown of blncRNA1. The y-axis represents enrichment of bound protein normalized to ChIP input. The control represents a motif matched CBS where CTCF has been shown not to bind [14].

To interrogate the function of blncRNA1, we engineered shRNA lentiviral expression vectors that target the 3’ or 5’ ends of the transcript. We confirmed knockdowns of blncRNA1 in IMR90s by RT-qPCR (Fig 4A, S1 File). If the transcript were linked to regulation of HOXA genes at CBS5, we would expect to see changes in the expression pattern of nearby HOXA genes. Upon knockdown of blncRNA, we observed significant up regulation of HOXA7 while other HOXA genes analyzed did not yield statistically significant changes (Fig 4B, S1 File).

### blncRNA1 Knockdown Results in Loss of Long-Range Interactions at the HOXA Locus

Changes in nearby HOXA genes upon blncRNA1 knockdown suggest that the transcript may be involved in long-range chromosomal interactions at the HOXA locus. Previous characterization of long-range interactions at CBS5 detected several interactions, upstream and downstream. Previously we have shown that knockdown of CTCF or Cohesin subunit RAD21 abolishes these long-range interactions at CBS5. To further characterize the role of blncRNA1 in higher order chromatin structure, we performed chromosome capture confirmation (3C) assays upon knockdown of blncRNA1 using the lentiviral vectors characterized in the previous section. We monitored the effects of knockdown on the long-range interactions that were previously published using CBS5 primer and primers specific to the upstream and down stream interaction sites [15]. Knockdown of blncRNA1 using shRNAs targeting the 5’ or 3’ of this transcript significantly reduced both upstream and downstream interactions at CBS5 (Fig 4C, S1 File). These results suggest that blncRNA1 may have a role in maintaining long-range interactions at the HOXA locus.

### blncRNA1 Knockdown Does Not Alter CTCF and Cohesin Interactions at CBS5

One explanation for reduction of long-range interactions at CBS5 upon blncRNA knockdown is that the transcript stabilizes CTCF-Cohesin interactions at CBS5. To test if blncRNA1 might participate in stabilizing CTCF-Cohesin interactions at CBS5, we assayed for occupancy of CTCF or Cohesin at CBS5 upon knockdown of blncRNA1. Surprisingly, knockdown of blncRNA1 did not affect CTCF or Cohesin occupancy at CBS5 (Fig 4D, S1 File). This result suggests that blncRNA1 may function independently of CTCF or Cohesin to facilitate or stabilize these long-range interactions at the HOXA locus detected by 3C. We also performed RNAPII ChIP-qPCR upon knockdown of blncRNA1 to monitor RNAPII occupancy at CBS5, to confirm that knockdown did not affect generation of the transcript itself. Occupancy of RNAPII at CBS5 was not affected upon knockdown of blncRNA1 (Fig 4D, S1 File).

In summary, CTCF and Cohesin at CBS5 are critical for the production of blncRNA1. blncRNA1 knockdown does not alter CTCF, Cohesin, or RNAPII interactions at CBS5. Thus, the generation of blncRNA1 is dependent on CTCF and Cohesin, but the transcript seems to contribute to stabilization of long-range interactions at the HOXA locus independently of Cohesin.

## Discussion

Here, we characterize CTCF-dependent promoter activity from a heterochromatin boundary element in the HOXA locus. This boundary element is an independently validated, evolutionarily conserved topological boundary element in mammalian genomes [11, 14]. RNAPII occupancy at this boundary element is consistent with the observed promoter activity in reporter assays. From these results, we were able to recover a long noncoding capped and polyadenylated transcript (blncRNA1) that emanates from the boundary element. We show that expression of this blncRNA1 depends on CTCF and its cognate site within the boundary element. We also observe that expression of blncRNA1 depends on Cohesin. The dependency of CTCF-Cohesin interactions on blncRNA1 expression has also been observed in RNA-seq data upon the knockdown of CTCF and Cohesin (S1 File, data not published). Perturbations of blncRNA1 by knockdowns resulted in loss of long-range chromosomal interactions. Long-range interactions at the HOXA locus blncRNA1 perturbations do not alter CTCF-Cohesin interactions at HOXA boundary element CBS5 thus suggesting this RNA has an integral role to long-range chromosomal interactions at the HOXA locus. The data presented suggests that long-range interactions at the HOXA locus are dependent on the expression of blncRNA1 resulting from CTCF-Cohesin interactions. Together, these results highlight a novel mechanism of maintaining higher order chromatin structure that involves CTCF, Cohesin and long noncoding RNA. Specifically several notable features of this mechanism are worth highlighting:

Our finding concerning promoter activity in a well-defined boundary element is analogous to the previous studies of HMR locus barrier element in *S*. *cerevisiae* and suggests that boundary activity is functionally conserved. Previous studies have shown that expression of tRNA^Thr^ was linked to the heterochromatin barrier activity at the HMR locus [18]. This barrier activity was further linked to interactions TFIIIB and TFIIIC that bind A-box and B-box elements, respectively. Interaction of these two factors is needed for transcription initiation of RNAPIII [18]. However, the exact nature of relationship between boundary and promoter activity remains unclear and requires additional studies to resolve. The presence of a promoter activity does not necessarily indicate a productive transcript. Potentially, the transcription machinery may function to provide or facilitate boundary activity. Alternatively, a nascent transcript made at a boundary may serve to create the heterochromatin boundary by mechanisms currently unknown for the mammalian genomes. In the case of *S*.*cerevisiae* the promoter activity was found to be more critical than the production of tRNA^Thr^.

Our initial survey of cell lines suggests that blncRNA1 is expressed in all the differentiated cells examined, but not in pluripotent embryonic stem cells, H1 and H9 lines. The lack of expression in ESC suggests that this transcript may have a function that is important for differentiated cells. Because we detected blncRNA1 expression in fibroblasts with the inverse patterns of H3K27me3 deposition at HOXA locus, we suspect that blncRNA1 function in higher order organization of HOXA locus regardless of the direction of the heterochromatin deposition relative to CBS5.

There are currently two mechanism in which noncoding transcripts are thought to stabilize chromosomal loops, indirectly through protein intermediaries or directly. In the case of the SRA transcript, it was shown that this transcript supports CTCF-Cohesin interactions at the H19/Igf2 locus [25]. Alternatively, the transcript may stabilize the loop via RNA-DNA interactions as seen with the *Kcqn1ot1* transcript that stabilizes the loop at the *Kcnq1* locus [26]. Although the exact mechanism of how blncRNA1 stabilizes chromosomal loops at the HOXA boundary is unclear, we were able to resolve that blncRNA1-mediated looping may be independent of CTCF-Cohesin interaction at CBS5. We demonstrate that blncRNA1 knockdowns resulted in loss of chromosomal looping but CTCF and Cohesin binding at the boundary was not affected. Thus, CTCF-Cohesin interactions are more than likely critical for generation of blncRNA1 and looping at CBS5 topological boundary involves CTCF, Cohesin and blncRNA1.

In summary, we have defined additional molecular mechanisms underlying a topological boundary element in the human genome. We find that promoter activity and generation of noncoding RNA from the boundary element may serve to stabilize topological organization of the locus. Thus, unexpectedly, we have discovered that transcription-promoting activity of CTCF is a key activity responsible for the generation of the noncoding RNA required for its function at the boundary element. Additional studies of other boundary associated long noncoding RNAs are required to examine generality of this mechanism, but the proposed mechanisms involving Cohesin and long non-coding RNAs are wholly consistent with the previous studies.

## Supporting Information

S1 Fig Reduced luciferase activity for initiator mutant CBS 5(-) reporter. The reporter signal is normalized to promoter activity detected in SV40 Promoter/Enhancer. The inset represents the predicted initiator sequence for the blncRNA1 gene and the mutant initiator sequence used in the reporter assay. The mutation in the initiator element resulted in significant reduction in reporter activity (*P* = 0.017, T-test)
(TIFF)

S2 Fig blncRNA1 expression in poster fibroblasts. (A—C) CTCF-RNAPII interactions in posterior fibroblasts. CTCF and RNAPII ChIP-qPCR were performed in fibroblasts from butt (A), leg (B), and butt-thigh (C). The y-axis represents enrichment of bound DNA normalized to the input. The control region is just outside the HOXA locus that has been shown not to bind CTCF. (D) blncRNA1 cDNA detection in posterior fibroblasts. blncRNA1 expression was analyzed in butt, leg, butt-thigh fibroblast. the relative expression levels were normalized to fetallung fibroblasts. (E) HOTAIR expression was used as a control to distinguish expression pattern of anterior and posterior fibroblasts. HOTAIR expression was normalized to foreskin fibroblast.
(TIFF)

S1 File All Supporting Tables. (Table A) Promoter Assay Primers. The genomic region highlighted in this table is from USC Genome Browser hg 19 build. The primer purpose specifies the cloning procedure needed to make constructs used in the reporter assays. (Table B) RACE Analysis and Isolation of blncRNA1. The primers in this table were used to detect blncRNA1 by RACE and for cloning of the full-length transcript. (Table C) blncRNA1 Expression Analysis Primers. The primers in this table were used to monitor expression of blncRNA1 by qRT-PCR. (Table D) Lentivirus Primers. The primers in this table were used in making the shRNA lentiviral constructs. (Table E) ChIP and 3C Primers. The primers in this table were used for ChIP and 3C analysis. The CBS5 and CBS Control primers were used for ChIP analysis; and the 3C Anchor, Upstream, and Downstream primer were used in 3C analysis. (Table F) Fetal Lung Fibroblast CTCF and RNAPII ChIP. The table shows the raw Ct values (Trial 1 Ct, Trial 2 Ct, and Trial 3 Ct), average Ct (Ave Ct) values, and enrichment fold (ChIP-Input) for each CTCF and RNAPII ChIP experiment. The top half of the chart represents CBS5 detection and the bottom half represents the CBS control. The replicates for each experiment (A, B, C) are also shown in this table. D) The overall analysis top half of the table is represents of the CTCF ChIP overall analysis and the bottom half represents the RNAPII ChIP overall analysis. The individual experimental averages, the average of all experiments (AVE Expts), and the standard deviation (STD) are included in this table. (Table G) blncRNA1 Expression in Human Embryonic Stem Cells. The table shows the raw Ct values (Trial 1 Ct, Trial 2, Ct, Trial 3 Ct), average Ct values (Ave Ct) for GAPDH expression (top half of table) and blncRNA1 expression (bottom half of table). The control was blncRNA1 expression in fetal lung fibroblast. blncRNA1 expression Ct values in H1 and H9 hESC were "Normalized to GAPDH" (GAPDH Ave Ct—blncRNA1 Ave Ct). The "Normalized to Control" was calculated by subtracting normalized GAPDH levels in H1 and H9 from the fetal lung fibroblast control. The "Molecules Normalized to Control" represents 2^(Normalized to Control). (Table H) blncRNA1 Expression in Posterior Fibroblast Lines. The table shows the raw Ct values (Trial 1 Ct, Trial 2, Ct, Trial 3 Ct), average Ct values (Ave Ct) for GAPDH expression (top of table), HOTAIR Expression (middle of table), and blncRNA1 expression (bottom of table). The "Normalized to GAPDH" (GAPDH Ave Ct—HOTAIR or blncRNA1 Ave Ct). The "Ct Normalized" was calculated by subtracting normalized GAPDH levels from foreskin (HOTAIR normalization) or fetal lung fibroblast (blncRNA1 normalization). The "Molecules Normalized" represents 2^(Ct Normalized). (Table I) CTCF and RNAPII ChIP in Posterior Fibroblast Cell Lines. The table shows the raw Ct values (Trial 1 Ct, Trial 2 Ct, and Trial 3 Ct), average Ct (Ave Ct) values, and enrichment fold (ChIP-Input) for each CTCF and RNAPII ChIP experiment. ChIP was performed for foreskin (A), leg (B), butt (C) and butt-thigh (D) fibroblast cell lines. (Table J) Effect of CTCF and Rad21 knockdowns on blncRNA1 expression. The table shows the raw Ct values (Trial 1 Ct, Trial 2, Ct, Trial 3 Ct), average Ct values (Ave Ct) of GAPDH, blncRNA1, CTCF, and Cohesin subunit Rad21. Expression levels for GAPDG, blncRNA1, CTCF, and Rad21 have measured under the PGIPZ control (top of the table), CTCF kd (middle of the table), and the Rad21 kd (bottom of the table). The "Normalized to GAPDH" represents Ct values normalized to GAPDH expression under each kd condition. The "Ct Normalized to PGIPZ" was calculated by subtracting normalized GAPDH levels under the CTCF kd or RAD 21 kd conditions from those calculated in the PGIPZ control. The "Molecules Normalized to Control" represents 2^(Ct Normalized to PGIPZ). (Table K) blncRNA1 3' KD target. The table shows the raw Ct values (Trial 1 Ct, Trial 2 Ct, Trial 3 Ct), average Ct Values (Ave Ct) for the shRNA524 kd of blncRNA1. GAPDH and blncRNA1 expression levels were measured and used to monitor knockdown efficiency. The "Normalized to GAPDH" represents Ct values normalized to GAPDH expression. The "Normalized to Control" represents Ct normalization to the shRNA negative control lentivirus. The "Molecules Normalized to Control" represents 2^(Ct Normalized to PGIPZ). The knockdown replicates are shown in tables (A-B) ad the overall analysis (C) is included. The standard deviation (STD) is also calculated in the overall analysis table. (Table L) blncRNA1 5' KD target. The table shows the raw Ct values (Trial 1 Ct, Trial 2 Ct, Trial 3 Ct), average Ct values (Ave Ct) for the shRNA37 kd of blncRNA1. GAPDH and blncRNA1 expression levels were measured and used to monitor knockdown efficiency. The "Normalized to GAPDH" represents Ct values normalized to GAPDH expression. The "Normalized to Control" represents Ct normalization to the shRNA negative control lentivirus. The "Molecules Normalized to Control" represents 2^(Ct Normalized to PGIPZ). The knockdown replicates are shown in tables (A-C) ad the overall analysis (D) is included. The standard deviation (STD) is also calculated in the overall analysis table. (Table M) HOXA Expression upon Control KD. The table shows the raw Ct values (Trial 1 Ct, Trial 2 Ct, Trial 3 Ct), average Ct values (Ave Ct), the copies, and number of molecules for HOXA6-A10 when using the shRNA control lentivirus. The replicates for each experiment (A-C), and the overall analysis (D), are also shown. The standard deviation (STD) is represented in the overall analysis. (Table N) HOXA Expression upon blncRNA1 KD. The table shows the raw Ct values (Trial 1 Ct, Trial 2 Ct, Trial 3 Ct), average Ct values (Ave Ct), the copies, and number of molecules for HOXA6-A10 when using the shRNA kd lentivirus. The replicates for each experiment (A-C), and the overall analysis (D), are also shown. The standard deviation (STD) is represented in the overall analysis. The p-Value shown in the overall analysis was calculated as a comparison to S13 Table using T-test. (Table O) Chromosome Capture Confirmation (3C) Analysis. The table shows the raw Ct Values (Trial 1 Ct, Trial 2 Ct, Trial 3 Ct, Trial 4 Ct, Trial 5 Ct, Trial 6 Ct), average Ct, interaction and final frequency for the lentivrial shRNA control, lentivrial 5' shRNA kd (shRNA37), and lentiviral 3' shRNA kd (shRNA 524). The BAC control for the 3C analysis is also shown in this figure. Replicates for each 3C experiment is shown (A-C) as well as the overall analysis and the standard deviation (STD) for the upstream (u10), and downstream (p40) positions. (Table P) CTCF Occupancy upon blncRNA1 KD. The table shows the raw Ct values (Trial 1 Ct, Trial 2 Ct, and Trial 3 Ct), average Ct (Ave Ct) values, and enrichment fold (ChIP-Input) for the CTCF ChIP experiment performed after kd of blncRNA1. The replicates for each experiment (A, B, C), ad the overall analysis (D) are also shown. The overall analysis includes averages of each experiments and the standard deviation (STD). (Table Q) RNAPII Occupancy upon blncRNA1 KD. The table shows the raw Ct values (Trial 1 Ct, Trial 2 Ct, and Trial 3 Ct), average Ct (Ave Ct) values, and enrichment fold (ChIP-Input) for the RNAPII ChIP experiment performed after kd of blncRNA1. The replicates for each experiment (A, B, C), ad the overall analysis (D) are also shown. The overall analysis includes averages of each experiments and the standard deviation (STD). (Table R) Cohesin Occupancy upon blncRNA1 KD. The table shows the raw Ct values (Trial 1 Ct, Trial 2 Ct, and Trial 3 Ct), average Ct (Ave Ct) values, and enrichment fold (ChIP-Input) for the Cohesin subunit Rad21 ChIP experiment performed after kd of blncRNA1. The replicates for each experiment (A, B, C), ad the overall analysis (D) are also shown. The overall analysis includes averages of each experiments and the standard deviation (STD).
(XLSX)
