# Supplementary figures and images for: Boundary Associated Long Noncoding RNA Mediates Long-Range Chromosomal Interactions
Source: PLoS One. 2015 Aug 24;10(8):e0136104. doi: 10.1371/journal.pone.0136104 (PMC4547746; doi:10.1371/journal.pone.0136104)

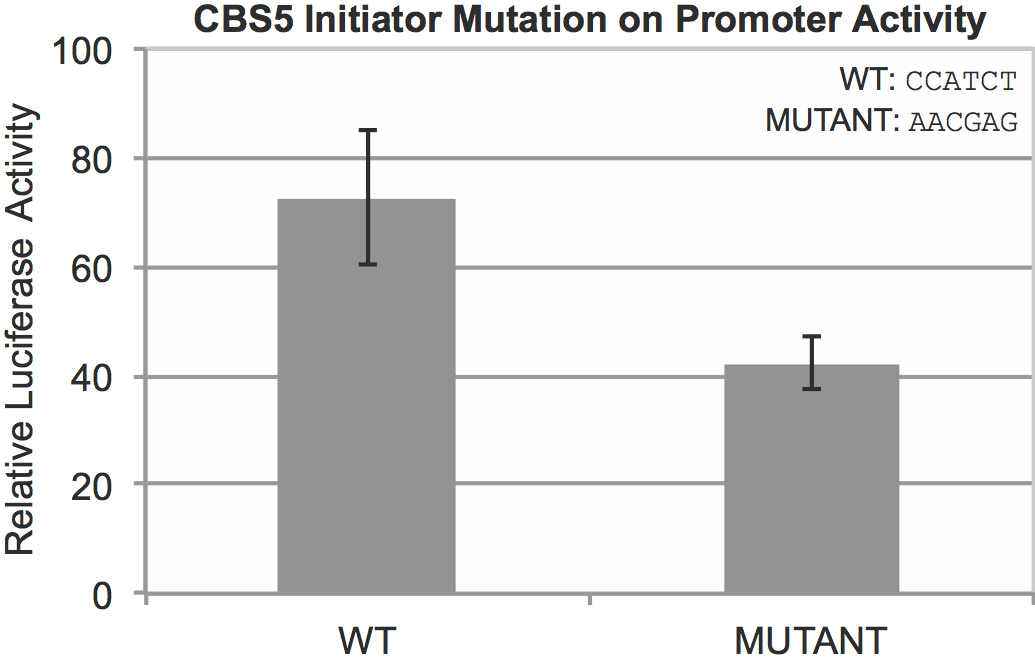

Supplement: S1 Fig — The reporter signal is normalized to promoter activity detected in SV40 Promoter/Enhancer. The inset represents the predicted initiator sequence for the blncRNA1 gene and the mutant initiator sequence used in the reporter assay. The mutation in the initiator element resulted in significant reduction in reporter activity (P = 0.017, T-test) (TIFF) [file pone.0136104.s001.tiff]

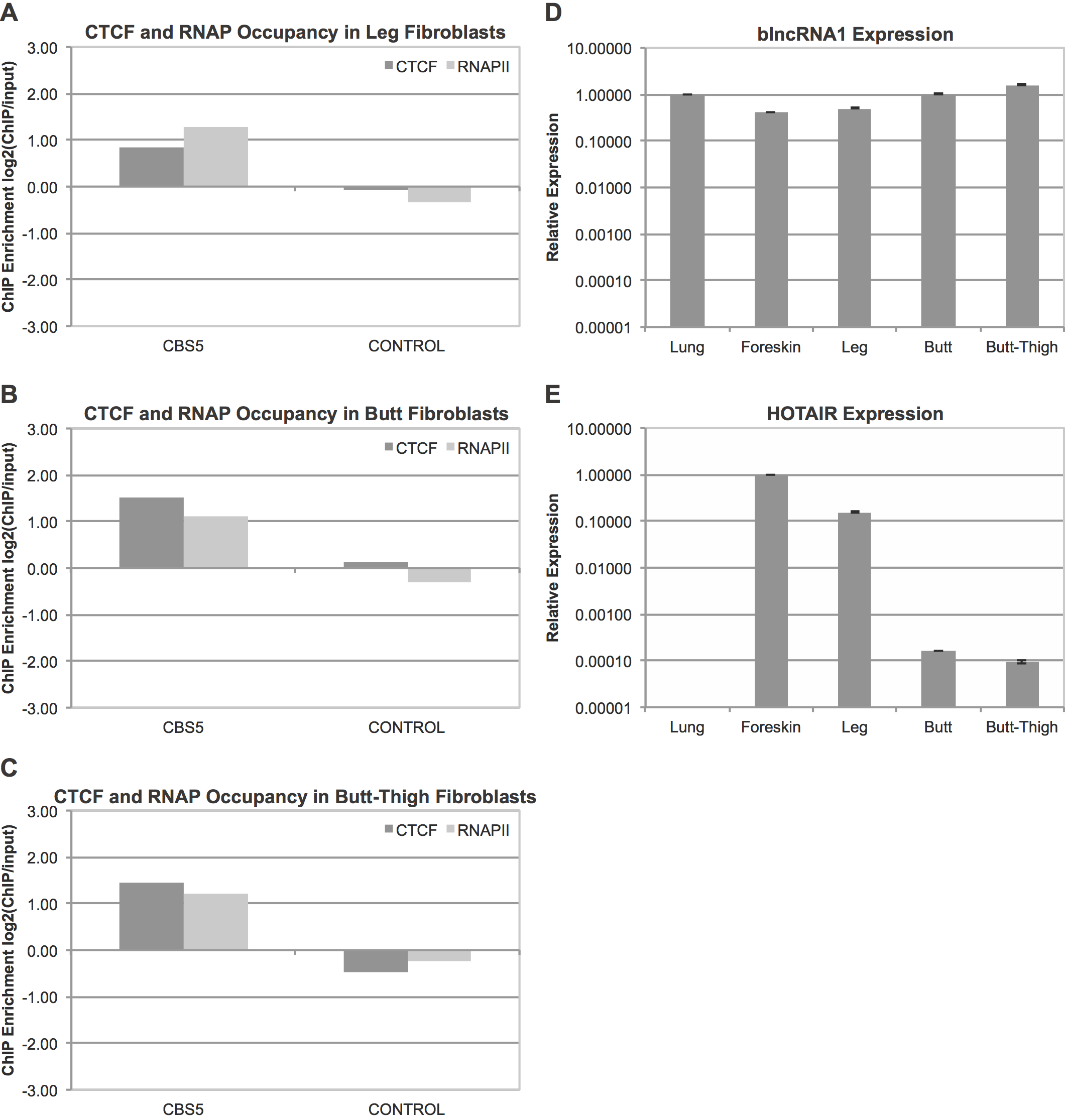

Supplement: S2 Fig — (A—C) CTCF-RNAPII interactions in posterior fibroblasts. CTCF and RNAPII ChIP-qPCR were performed in fibroblasts from butt (A), leg (B), and butt-thigh (C). The y-axis represents enrichment of bound DNA normalized to the input. The control region is just outside the HOXA locus that has been shown not to bind CTCF. (D) blncRNA1 cDNA detection in posterior fibroblasts. blncRNA1 expression was analyzed in butt, leg, butt-thigh fibroblast. the relative expression levels were normalized to fetallung fibroblasts. (E) HOTAIR expression was used as a control to distinguish expression pattern of anterior and posterior fibroblasts. HOTAIR expression was normalized to foreskin fibroblast. (TIFF) [file pone.0136104.s002.tiff]
